# The role of contour polarity, objectness, and regularities in haptic and visual perception

**DOI:** 10.3758/s13414-018-1499-6

**Published:** 2018-03-16

**Authors:** Stefano Cecchetto, Rebecca Lawson

**Affiliations:** Institute of Psychology, Health and Society, Eleanor Rathbone Building, University of Liverpool, Bedford Street South, Liverpool, L69 7ZA UK

**Keywords:** Regularities, Symmetry, Repetition, Modality, Concavity, Convexity, Figure-ground, Part decomposition

## Abstract

Regularities like symmetry (mirror reflection) and repetition (translation) play an important role in both visual and haptic (active touch) shape perception. Altering figure-ground factors to change what is perceived as an object influences regularity detection. For vision, symmetry is usually easier to detect within one object, whereas repetition is easier to detect across two objects. For haptics, we have not found this interaction between regularity type and objectness (Cecchetto & Lawson, *Journal of Experimental Psychology: Human Perception and Performance, 43,* 103–125, 2017; Lawson, Ajvani, & Cecchetto, *Experimental Psychology, 63,* 197–214, 2016). However, our studies used repetition stimuli with mismatched concavities, convexities, and luminance, and so had mismatched contour polarities. Such stimuli may be processed differently to stimuli with matching contour polarities. We investigated this possibility. For haptics, speeded symmetry and repetition detection for novel, planar shapes was similar. Performance deteriorated strikingly if contour polarity mismatched (keeping objectness constant), whilst there was a modest disadvantage for between-2objects:facing-sides compared to within-1object:outer-sides comparisons (keeping contour polarity constant). For the same task for vision, symmetry detection was similar to haptics (strong costs for mismatched contour polarity, weaker costs for between-2objects:facing-sides comparisons), but repetition detection was very different (weak costs for mismatched contour polarity, strong benefits for between-2objects:facing-sides comparisons). Thus, objectness was less influential than contour polarity for both haptic and visual symmetry detection, and for haptic repetition detection. However, for visual repetition detection, objectness effects reversed direction (within-1object:outer-sides comparisons were harder) and were stronger than contour polarity effects. This pattern of results suggests that regularity detection reflects information extraction as well as regularity distributions in the physical world.

Regularities like symmetry (mirror reflection) and repetition (translation) are ubiquitous in our environment and provide important visual cues that we use to structure and organize information into meaningful elements (Palmer, 1989; Wagemans, 1995). Vision scientists have long striven to understand how and why regularities are detected so efficiently by humans (for reviews, see Leeuwenberg, 2010; Treder, 2010; Tyler, 1995; van der Helm, 2014; Wagemans, 1995, 1997). Symmetry is known to provide a major grouping principle for the representation of visual shape (Palmer, 1989; Royer, 1981; van der Helm & Leeuwenberg, 1996), for figure-ground segregation (Baylis & Driver, 2001; Driver, Baylis & Rafal, 1992; Machilsen, Pauwels, & Wagemans, 2009), amodal completion (Kanizsa, 1985; van Lier, van der Helm, & Leeuwenberg, 1995), and object recognition (Pashler, 1990; Vetter & Poggio, 1994). It has been argued that the powerful and wide-ranging influence of regularities on perceptual processing may arise because symmetry and repetition in the 2-D visual input provide us with important, proximal cues to nonaccidental, distal properties of our 3-D physical environment (Baylis & Driver, 1995). Specifically, as discussed below, symmetry may be used to signal the presence of a single object, and repetition used to indicate the presence of multiple, similarly shaped objects (Cecchetto & Lawson, 2017).

The terminology used by researchers to describe regularities is not consistent. Here, we will discuss only two types of *regularity*: bilateral, mirror-reflectional symmetry, that we will refer to as *symmetry*, and translational symmetry, that we will term *repetition*. In the present study, we asked people to detect regular from irregular (random) stimuli. Regularities occurred across pairs of *critical contours*. These contours consisted of the outer left and right sides of a single object (henceforth *within-1object:outer-sides* stimuli), the two facing sides of two objects (henceforth *between-2objects:facing-sides* stimuli; here the contours flanked an empty, background space) or the two right sides of two objects (henceforth *between-2objects:right-sides* stimuli; here, the contours flanked a background space and one of two objects; see Fig. 1). *Contour polarity* refers to the polarity of features associated with the side of a contour owned by an object (as opposed to the background side). It includes contour luminance polarity (whether the object region next to the contour is dark or light) and contour curvature polarity (whether the local contour is concave or convex). A pair of contours have *matching contour polarity* if any relevant features are identical at equivalent locations along them and they have mismatching contour polarity if the features differ (see Fig. 1 for examples).

**Fig. 1:**
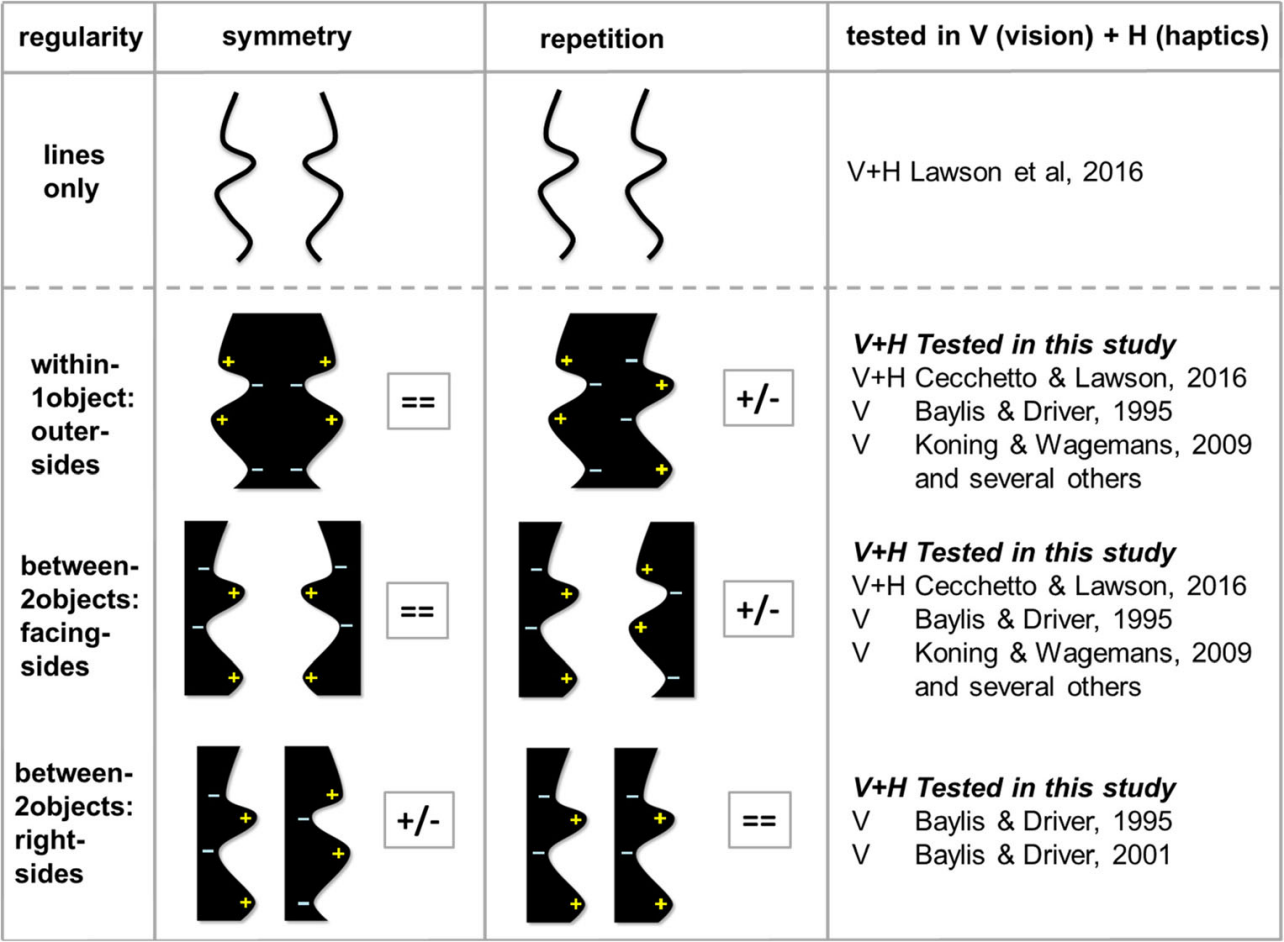
Examples of symmetrical (left) and repeated (right) regular stimuli. Irregular stimuli are not shown here, but they were identical to the regular stimuli except that the left and right critical contours were created from two different, unique lines. Top row: Pairs of lines which are not bound to surfaces, similar to the stimuli used by Lawson, Ajvani, and Cecchetto (2016). Second and third rows: Same pairs of lines used to create the outline contours of within-1object:outer-sides and between-2objects:facing-sides stimuli, similar to the stimuli used by Cecchetto and Lawson (2017). Bottom row: The same pairs of lines incorporated into between-2objects:right-sides stimuli. Stimuli with matching contour polarity are labelled = =. Here, there was the same colour and luminance in the object region bounded by these critical contours, and convexities (+) and concavities (−) matched at equivalent locations along these contours. Stimuli with mismatching contour polarity are labelled +/−. These had opposite polarities for colour, luminance, and convexities and concavities along the critical contours. Several regularity detection studies (e.g., Bertamini, 2010; Bertamini, Friedenberg & Kubovy, 1997; Cecchetto & Lawson, 2017; Koning & Wagemans, 2009) used the within-1object:outer-sides and between-2objects:facing-sides stimuli whilst Baylis and Driver (2001) used the between-2objects:right-sides stimuli. However, as far as we are aware, only Baylis and Driver (1995) have previously used the six stimulus conditions that were tested in the present study (i.e., all three lower rows of stimuli shown here), and they only tested visual (not haptic) regularity detection

Visual symmetry is usually easier to detect than other regularities such as repetition (Baylis & Driver, 1994, 1995; Mach, 1886/1959) and seems to have greater salience than repetition (Treder & van der Helm, 2007). Understanding why and when symmetry detection is often efficient requires explaining the well-established interaction between regularity type and objectness (Koning & Wagemans 2009; see also Baylis & Driver, 1995, 2001; Bertamini, Friedenberg, & Kubovy, 1997; Cecchetto & Lawson, 2017; Lawson et al., 2016). The exact nature of this interaction varies across different studies (Koning & Wagemans, 2009). However, in general, symmetry detection is better for within-1object:outer-sides stimuli than for between-2objects:facing-sides stimuli, whereas the reverse is true for repetition detection. These results cannot be explained by effects of contour polarity and parts decomposition alone (Baylis & Driver, 1995; Hoffman & Richards, 1984) because these are the same for within-1object:outer-sides and between-2objects:facing-sides stimuli (see rows 2 and 3 of Fig. 1, respectively). Instead, some other factor must be driving the interaction. Koning and Wagemans (2009; see also Cecchetto & Lawson, 2017; Lawson et al., 2016) argued that the Regularity Type × Objectness interaction might arise because symmetry and repetition provide different cues about the world. Visual regularities may provide important information about how to segment a scene into objects, with symmetry used to signal the presence of a single object, and repetition used to indicate the presence of multiple, similarly shaped objects (Cecchetto & Lawson, 2017). This could explain why symmetry is easier to detect within a single object whilst repetition is easier to detect across different objects.

An important limitation with most studies that have investigated the interaction between regularity type and objectness (e.g., Bertamini, 2010; Bertamini et al., 1997; Cecchetto & Lawson, 2017; Koning & Wagemans, 2009; Lawson et al., 2016) is that the planar stimuli they used confounded regularity type and contour polarity. Specifically, symmetrical stimuli usually had matching contour polarity so were truly regular, but the repetition stimuli had mismatching contour polarity (with respect to colour, luminance, and/or concavities and convexities) and so might be best described as antiregular (compare the within-1object:outer-sides and between-2objects:facing-sides stimuli in rows 2 and 3 of Fig. 1). Van der Helm and Treder (2009) found evidence that the visual system treats regularities differently depending on whether contour polarity matched or mismatched. They argued that only the dot stimuli used by Corballis and Roldan (1974) and Treder and van der Helm (2007) presented repetition stimuli with matching contour polarity to investigate the Regularity Type × Objectness interaction. Corballis and Roldan (1974) used pairs of dot patterns that were either shown next to each other (so they could be perceived as a single object) or separated by a gap (so they could be perceived as two distinct objects). Treder and van der Helm (2007) used symmetrical and repeated dot patterns presented stereoscopically. They relied on grouping principles to ensure that the dots were perceived as a single object (because they lay on the same depth plane) or two distinct objects (because the dots lay on two different depth planes). In both studies, the interaction (symmetry detection being easier for within-1object:outer-sides compared with between-2objects:facing-sides stimuli, and vice versa for repetition detection) was found only for dot stimuli, and in neither study was a clear, between-2objects:facing-sides advantage found for repetition. In summary, there is little evidence that repetition with matching contour polarity (as opposed to with mismatching contour polarity) is easier to detect visually. This, in turn, means that the Regularity Type × Objectness interaction could be driven by either a difference in objectness or a difference in contour polarity.

One way to progress our understanding of how and why we are sensitive to symmetry and repetition is to find a new approach to test regularity detection. To achieve this, we have investigated a different modality—namely, haptics—our sense of active touch. Vision and haptics extract information from similar environments and share many processing goals. If effects on regularity detection generalise across these two modalities, then this would suggest that these effects arise because regularities provide important cues about objects in our external, physical world. However, if effects on regularity detection are modality specific, this would indicate that these effects reflect stimulus exploration and information extraction and storage. Compared with research on visual regularity detection, there has been relatively little research investigating the haptic perception of symmetry (for a recent review, see Cattaneo et al., 2014). It is well established that haptics can detect symmetry but, as far as we are aware, we are the only researchers to have established that haptics can also detect repetition (Cecchetto & Lawson, 2017; Lawson et al., 2016).

In order to understand the role of regularity detection in object perception, we have contrasted regularity detection in vision and in haptics by manipulating several potential cues to objectness such as regularity type (symmetry vs. repetition) and line separation. For vision we replicated the Regularity Type × Objectness interaction previously found, but for haptics we found no effect of objectness for either symmetry or repetition detection (Cecchetto & Lawson, 2017; Lawson et al., 2016; Lawson & Cecchetto, 2018) for stimuli with the axis of regularity aligned with the body midline. In these experiments, the same 3-D objects generated the input stimuli for vision and for haptics. Thus, this modality-specific difference in the effects of objectness provides evidence that regularity detection does not solely reflect external properties of our physical environment.

As discussed above, one concern raised by van der Helm and colleagues about most studies investigating regularity detection is that they confounded effects of regularity type and contour polarity. Lawson et al. (2016) addressed this issue by comparing the haptic and visual detection of regularities for pairs of lines with small, medium, or large separations (see the top row of Fig. 1). Such stimuli avoid the problems of mismatching contour polarity because they have no surfaces, so contour polarity cannot mismatch for colour or luminance whilst concavities and convexities cannot be defined unambiguously for line-only stimuli. We predicted that lines with small separations were more likely to be grouped together and perceived as belonging to a single object, whilst well-separated lines would not be grouped together and would be perceived as belonging to two different objects. If so, then the effects of line separation should interact with those of regularity type: If symmetry is used as a cue for the presence of a single object, then it should be easier to detect with small line separations, and if repetition is used as a cue for the presence of multiple, similarly-shaped objects, then it should be easier to detect with large line separations. For vision, as predicted, increased line separation disrupted symmetry detection more than repetition detection. However, for haptics, symmetry and repetition detection were similarly disrupted by increased line separation. Thus, the interaction between regularity type and objectness found for vision did not generalise to haptics. Both findings were consistent with the results reported by Cecchetto and Lawson (2017) for closed-contour, planar shapes.

However, there remains an important concern with the repetition stimuli that have been claimed to vary objectness without introducing a confound of mismatching contour polarities (Corballis & Roldan, 1974; Lawson et al., 2016; Treder & van der Helm, 2007). The concern is that these stimuli (small sets of dots or pairs of lines) might not be considered to be objects at all. It is difficult to formally define what is an object (Feldman, 2003) and researchers claiming to manipulate what is perceived as an object often fail to justify their choice of stimuli. Nevertheless, stimuli comprising dots or lines lack many of the features that are typical of everyday objects, such as having closed contours and solid surfaces. Worse, such stimuli may be trapped in a paradoxical situation. If they are *not* interpreted as objects then surely they are not suitable stimuli to use to investigate objectness. However, if they *are* perceived as objects then, arguably, that is because they are perceived as having something like a contour-bounded shape (e.g., created by joining adjacent dots or by connecting the nearest ends of lines together). People can behave as if contours are present when they do not objectively exist, for example in illusions involving amodal completion such as Kanizsa’s (1976) triangle. If the stimuli used by Corballis and Roldan (1974), Lawson et al., (2016), and Treder and van der Helm (2007) were perceived as contour-bounded shapes then these contours would have polarities defined by concavities and convexities. We do not assume that objectness is an all-or-nothing property of stimuli. Instead, we think that multiple cues combine to determine the extent to which a stimulus is perceived as an object. This means that the dot and line stimuli used by Corballis and Roldan (1974), Lawson et al. (2016), and Treder and van der Helm (2007) may have object-like qualities. This, in turn, means that these stimuli could have suffered from the same confound between regularity type and contour polarity that we discussed above. The goal of the present study was to avoid these confounds by independently assessing the role of matching versus mismatching contour polarity and the role of objectness on regularity detection using planar shapes with well-defined bounding contours.

In summary, in the present study, we aimed to tease apart the roles of contour polarity and objectness by comparing regularity detection for a new set of between-2objects:right-sides stimuli (see the bottom row of Fig. 1), in addition to the within-1object:outer-sides and between-2objects:facing-sides stimuli used in our previous studies. The within-1object:outer-sides conditions had matching contour polarity for symmetry and mismatching contour polarity for repetition. Across the four between-2objects conditions, there was both matching and mismatching contour polarity for both symmetry and repetition. We compared regularity detection by haptics (Experiment 1) and by vision (Experiment 2). In each experiment we focussed on two comparisons. First, we investigated the role of objectness whilst holding contour polarity constant, by comparing regularity detection for within-1object:outer-sides and between-2objects conditions. For the between-2objects conditions, symmetry detection was tested using between-2objects:right-sides stimuli which had matching contour polarity, whilst repetition detection was tested using between-2objects:facing-sides stimuli which had mismatching contour polarity. Second, we investigated the role of contour polarity whilst holding objectness constant, by comparing stimuli with matched versus mismatched contour polarities. This was done by comparing regularity detection for between-2objects:facing-sides and between-2objects:right-sides stimuli. We conducted these two separate comparisons because, for our shapes, it was not possible to fully cross the factors of objectness (one for within-1object:outer-sides vs. two for between-2objects:facing-sides and between-2objects:facing-sides stimuli) and contour polarity (matching or mismatching).

## Experiment 1

Participants haptically explored unseen, planar objects and decided if they had two regular contours. The objects were defined by being smooth plastic shapes that were raised 5 mm above a cardboard background. Symmetry detection and repetition detection were tested in separate blocks. For regularity, we expected to find no interaction between regularity type and objectness, as reported by Cecchetto and Lawson (2017). As far as we are aware, the effects of contour polarity have not been investigated for haptics.

### Method

#### Participants

There were 24 participants (16 females, mean age = 20 years, *SD* = 4.5 years, range: 18–40). They were either volunteers or undergraduate students from the University of Liverpool, who participated for course credit, and who reported no known conditions affecting their sense of touch. All participants completed the Edinburgh Handedness Inventory, that revealed two left-handers, one female and one male (mean score = 91.7, range: −100–100). Both the experiments reported here received ethical approval from the local ethics committee.

#### Materials and design

A laser cutter was used to produce the stimuli from 5-mm thick black acrylic sheets. Each stimulus included two critical contours, each of which was defined by the same unique line (for regular stimuli) or two different unique lines (for irregular stimuli). Twelve stimuli (regular/irregular × symmetry/repetition × within-1object:outer-sides/between-2objects:facing-sides/between-2objects:right-sides) were created from each of 20 unique lines to produce a set of 240 stimuli. All six regular stimuli created from a given unique line included the same two critical contours. The same was true for all six irregular stimuli created from that unique line. Only contour polarity (defined by the location of surfaces) and the nature of the regularity (symmetry or repetition) changed across each subset of six stimuli. A surface lay between the two critical contours for within-1object:outer-sides stimuli, surfaces were on the outside of the two contours for between-2objects:facing-sides stimuli, and surfaces were on the left side of each contour for between-2objects:right-sides stimuli (see Fig. 1).

The 240 stimuli were each glued onto a 10 cm × 10 cm brown cardboard base. The unique lines each had four vertices and were a subset of those used by Cecchetto and Lawson (2017). They were chosen by ordering our previous set of 40 unique lines by the overall accuracy of regularity detection for each line, then selecting alternate lines so the lines used spanned the range of difficulty. Further details about the creation of the unique lines are given in Cecchetto and Lawson (2017) and Lawson et al. (2016). Cecchetto and Lawson (2017) used unique lines with straight segments only. Here, the lines were smoothed to give rounded vertices to ensure that the participant’s fingers could feel around them.

The 240 stimuli were divided into two equal subsets. Each participant was presented with one subset. Within this subset, each of the 20 unique lines appeared as the left critical contour three times for symmetrical stimuli (once per stimulus condition) and three times for repetition stimuli (once per stimulus condition). Participants completed two blocks of 60 trials, one testing symmetry detection and the other testing repetition detection. Within each block, half the stimuli were regular and half were irregular, with 10 of each type from each stimulus condition (within-1object:outer-sides, between-2objects:facing-sides, and between-2objects:right-sides). Trials were presented in a fixed, pseudorandom order. Half of the participants detected symmetry first, and the remainder detected repetition first. Six participants from each of these two groups were assigned to each of the two stimulus subsets.

**Fig. 2:**
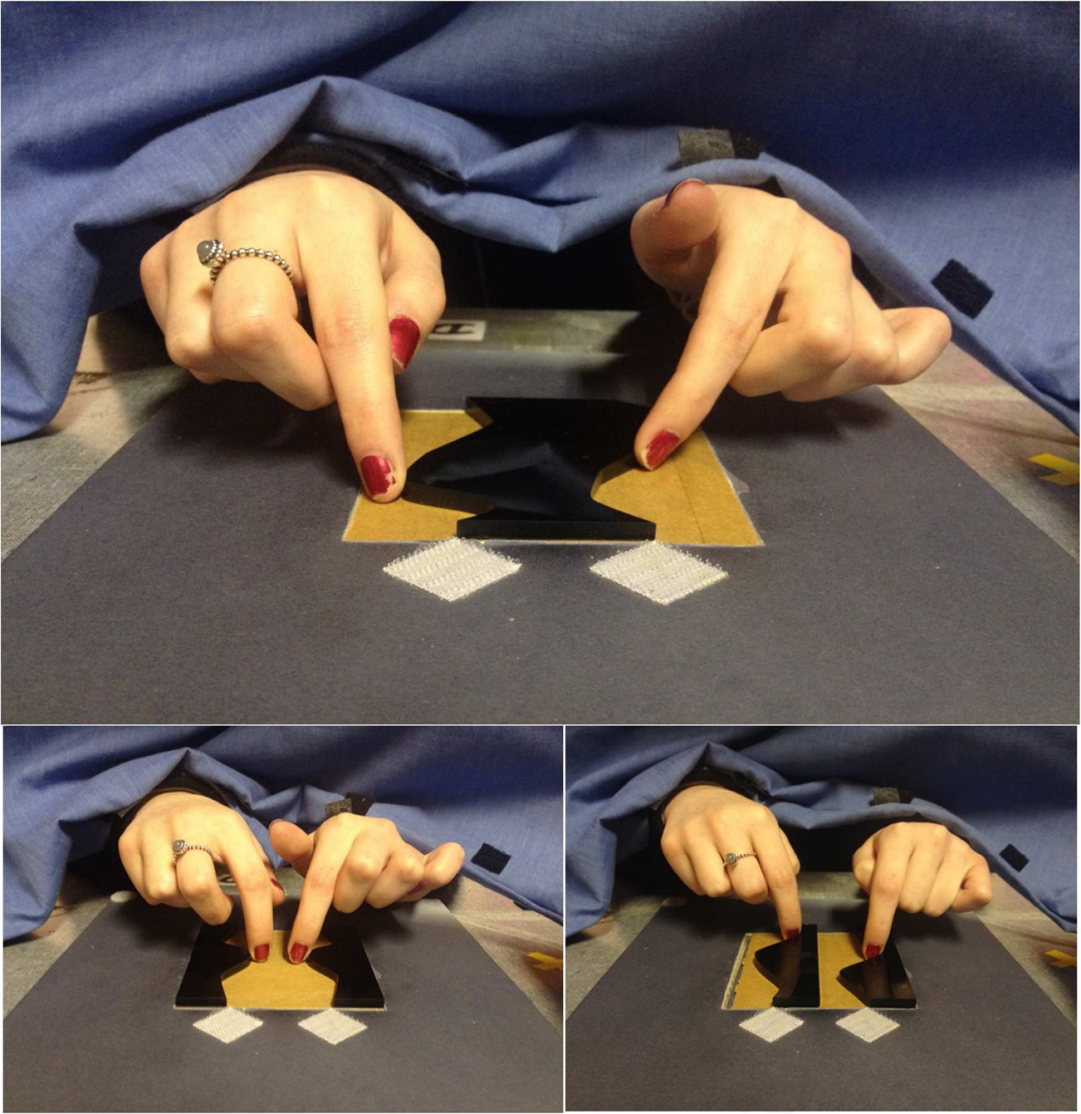
Examples of haptic exploration of an irregular, within-1object:outer-sides stimulus (top), a symmetrical, between-2objects:facing-sides stimulus (bottom left), and an irregular, between-2objects:right-sides stimulus (bottom right) in Experiment 1, as seen from the experimenter’s point of view. Two white diamond patches marked the rest positions and were located above the top of each of the critical contours of the stimulus

Participants sat in a normally lit lab behind a 70-cm high table. A thick curtain hung in front of the table, blocking their view of the stimulus and their hands (see Fig. 2). Participants responded using one of two foot pedals. On the table in front of the curtain there were two labels, “same” on the left and “different” on the right, to remind participants which foot pedal they should use to respond to regular and irregular stimuli, respectively. Participants were told to centre their body midline with the midpoint of the two response labels and the two foot pedals. Stimuli were placed with the nearest side 20 cm from the edge of the table and approximately 45 cm from the participant’s body. Stimuli were slotted into a fixed foam-board frame with a 10.1 cm × 10.1 cm aperture (see Fig. 2). The frame prevented the stimuli from moving during haptic exploration. Stimuli were always presented with the axis of regularity of the critical contours aligned with the participant’s body midline. Two white, textured patches were placed above the top of each of the critical contours to mark the resting positions for each index finger, and to ensure that the critical contours were easy to locate. The centres of the patches were 5 cm apart.

#### Procedure

Prior to starting the experiment participants were told about the regularity type (symmetry or repetition) that they had to detect in the first block. They were then visually shown six examples of the type of stimuli that they were about to feel (one regular and one irregular for each of the three stimulus conditions). These stimuli were similar to the experimental stimuli but they were not included in the experimental set. Participants then performed six practise trials feeling each of the practise stimuli in turn. They were told to respond as quickly and accurately as possible, to explore the two critical contours simultaneously, to use one index finger to feel each critical contour, and not to rotate, move, or pick up the stimuli.

At the start of each trial the experimenter placed a stimulus in the frame whilst the participant kept their hands on the resting position patches for each hand (see Fig. 2). The experimenter then triggered an auditory go signal from the computer that indicated that the participant could move their hands from the resting positions to feel the stimulus. Reaction times were measured from the offset of the go signal until the participant responded by pressing the foot pedal. This triggered a high-pitch or a low-pitch feedback sound that indicated whether their response was correct or wrong respectively. The first experimental block began immediately after the six practise trials. At the end of this block participants were told about the new type of regularity that they would have to detect and they were visually shown six new practise stimuli. They then did six practise trials followed by the second block. Finally, participants were asked whether they had seen any of the stimuli. The experiment took about 50 minutes.

### Results

No participants were replaced and none reported that they had seen any of the stimuli. Analyses of variance (ANOVAs) were conducted on the mean correct reaction times (RT) and percentage of errors for regular trials only, and on sensitivity (*d'*) for all trials. Correct RT faster than 1 s or slower than 35 s were discarded as errors (less than 1% of trials). In the ANOVAs there were two within-participants factors: regularity type (symmetry or repetition) and condition (within-1object:outer-sides, between-2objects:facing-sides or between-2objects:right-sides). All pairwise differences noted below were significant (*p* < .05) in post hoc Newman–Keuls analyses. Appendix 1 gives the full ANOVAs for RT, errors and sensitivity (*d'*). Here, we focus on the theoretically important effects so we only report the results for the interaction of Regularity Type × Condition and the results for the two critical comparisons.

**Fig. 3:**
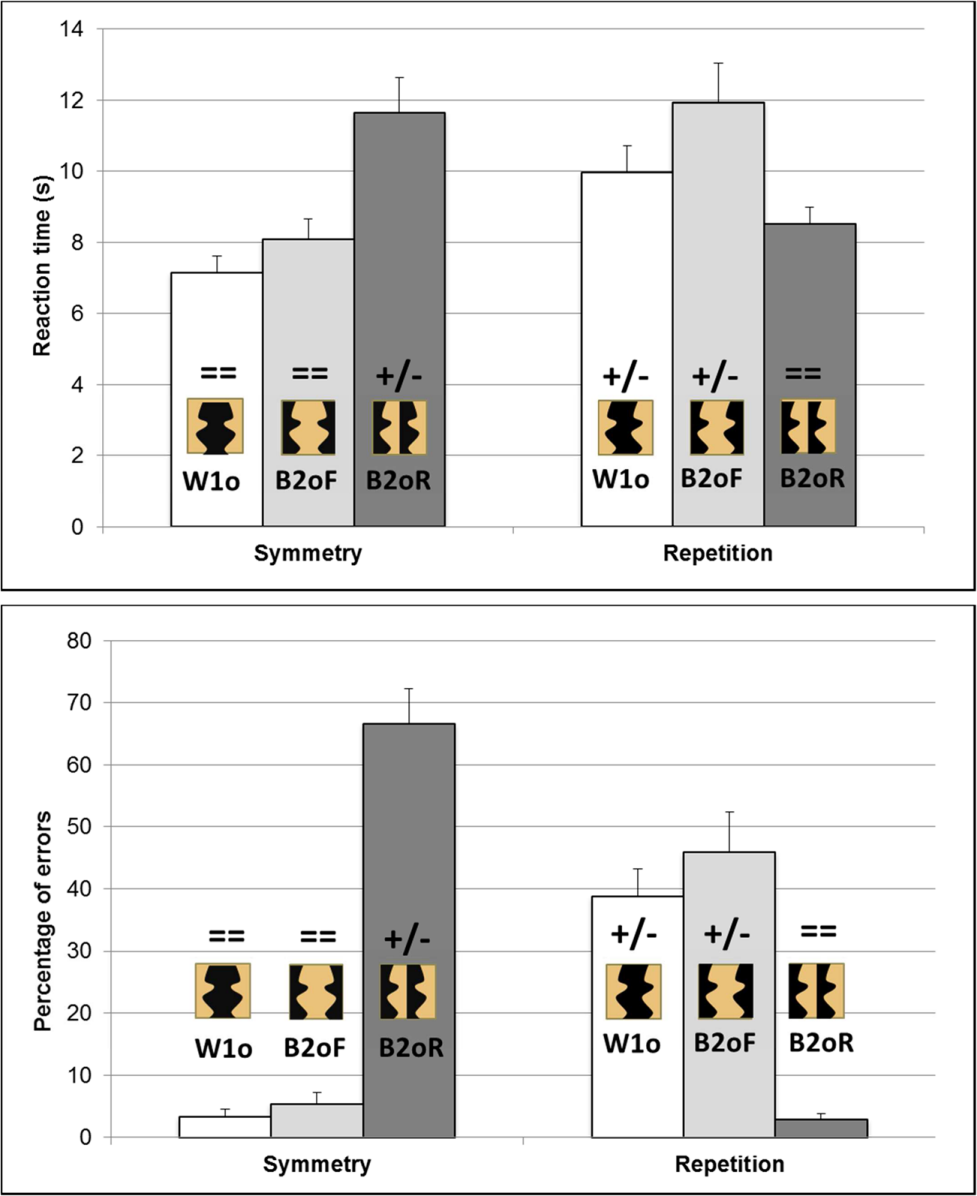
Results in Experiment 1, for regular trials, for the haptic detection of symmetry and repetition, for within-1object:outer-sides (W1o; white bars), between-2objects:facing-sides (B2oF; light-grey bars) and between-2objects:right-sides (B2OR; dark-grey bars) stimuli for RT (top), and errors (bottom). Example stimuli from each condition are shown on or above each bar, with a black object against a light brown background. Above each example stimulus, symbols indicate whether contour polarity across the pairs of critical contours matched (= =) or mismatched (+/−). Error bars represent one standard error of the mean

The interaction of Regularity Type × Condition was significant for RT, *F*(2, 46)= 34.86, *p* < .001, partial η^2^ = .60; errors, *F*(2, 46) = 90.47, *p* < .001, partial η^2^ = .79; and sensitivity, *F*(2, 46) = 28.10, *p* < .001, partial η^2^ = .55 (see Fig. 3).

First, we considered whether objectness influenced haptic regularity detection when contour polarity was held constant, by comparing performance for within-1object:outer-sides to between-2objects:facing-sides stimuli. For matching contour polarity stimuli, there was no significant difference in detecting symmetry for within-1object (7.1 s, 3%, *d'* of 2.08) versus between-2objects (8.1 s, 5%, 1.87) stimuli. For mismatching contour polarity stimuli, repetition was detected faster, but no more accurately or more sensitively, for within-1object (10.0 s, 39%, 1.05) versus between-2objects (11.9 s, 46%, 0.95) stimuli. Thus, the overall trend was for a weak within-1object:outer-sides advantage over between-2objects:facing-sides stimuli for both symmetry and repetition detection. However, this difference was only significant for RT for repetition.

Second, we investigated whether contour polarity influenced haptic regularity detection when objectness was held constant (since the critical contours always belonged to two different objects; see Fig. 1), by comparing performance for between-2objects:facing-sides to between-2objects:right-sides stimuli. Symmetry was detected faster, more accurately and more sensitively for matching contour polarity, facing-sides stimuli (8.1s, 5%, 1.87) than for mismatching contour polarity, right-sides stimuli (11.7s, 67%, 0.19). Indeed, people were unable to detect symmetry in the mismatching contour polarity condition, with 67% wrong “irregular” responses for regular trials versus 73% correct “irregular” responses for irregular trials. Repetition was detected faster and more accurately for matching contour polarity, right-sides stimuli (8.5 s, 3%, 1.20) than for mismatching contour polarity, facing-sides stimuli (11.9 s, 46%, 0.95), with the same trend for sensitivity but this latter difference was not significant in post hoc Newman–Keuls analyses. Thus, for both symmetry and repetition, regularities were much easier to detect if contour polarity matched rather than mismatched.

### Discussion

In Experiment 1, we compared the haptic detection of regularities for closed-contour, planar shapes (see Fig. 1). We investigated the role of objectness (one vs. two objects) and the role of contour polarity (matched vs. mismatched concavities and convexities) in the perception of symmetry and repetition. First, we found little influence of varying objectness when contour polarity was held constant. Performance was similar whether pairs of critical contours belonged to a single object (for the within-1object:outer-sides stimuli) or to two objects (for the between-2objects:facing-sides stimuli). For these comparisons, contour polarity always matched for symmetry detection and always mismatched for repetition detection (see Fig. 1). Overall, there was a modest within-1object:outer-sides advantage but this was only significant when comparing the speed of repetition detection. Crucially, the trends were similar for symmetry detection and repetition detection, so there was no Objectness × Regularity Type interaction. The results here replicated and extended our previous haptic findings (Cecchetto & Lawson, 2017; see also Lawson et al., 2016). We have consistently found no advantage for detecting repetition when it occurs between two objects rather than within a single object. This is in stark contrast to the results that we have obtained for the same task with the same stimuli for vision.

Second, we investigated the role of contour polarity by comparing stimuli with matched to mismatched concavities and convexities when objectness was held constant (by considering only stimuli in which the critical contours always belonged to two different objects; see Fig. 1). Between-2objects:right-sides stimuli produced a strikingly different pattern of performance to between-2objects:facing-sides stimuli, with opposite effects depending on the type of regularity being detected (see Fig. 3). Symmetry was much harder to detect for between-2objects:right-sides stimuli (here, contour polarity mismatched). Repetition was much harder to detect for between-2objects:facing-sides stimuli (here, again, contour polarity mismatched). These results demonstrate for the first time that contour polarity plays a crucial role in haptic shape perception.

## Experiment 2

In Experiment 2 we investigated whether different effects on regularity detection would be found for vision than we found for haptics. In Experiment 1, for haptics, no interaction was found between objectness and regularity type, in contrast to previous findings for vision. In addition, we found a new result, a powerful advantage for detecting haptic regularities if contour polarity matched. Experiment 2 largely replicated Experiment 1, except that the stimuli were presented visually, as pictorial images on a vertical monitor, rather than haptically, as 3-D, planar shapes. The objects were defined by being smooth, solid green surfaces that were presented on a background of random, black-and-white flickering noise. We presented the same conditions as in Experiment 1 (regular/irregular × symmetry repetition × within-1object:outer-sides/between-2objects:facing-sides/between-2objects:right-sides) to the same participants. Experiment 2 replicated the visual regularity detection conditions tested by Baylis and Driver (1995; see also Fig. 1), though many of the details of the design, task and stimuli differed.

First, we investigated the role of objectness when contour polarity was held constant. Unlike for haptics, we predicted that we would obtain a Regularity Type × Objectness interaction, consistent with previous results for vision (e.g., Baylis & Driver, 1995; Bertamini et al., 1997; Cecchetto & Lawson, 2017; Koning & Wagemans, 2009; Lawson et al., 2016). We expected to find a within-1object:outer-sides advantage for symmetry detection, but a between-2objects:facing-sides advantage for repetition detection. This would be consistent with symmetry being used as a cue to the presence of a single object in the external, physical world and repetition being associated with the presence of multiple, similarly shaped objects (Cecchetto & Lawson, 2017; Lawson et al., 2016).

Second, we investigated the role of contour polarity when objectness was held constant. We predicted that, as for haptics in Experiment 1, regularities would be harder to detect if they had mismatching rather than matching contour polarities (van der Helm & Treder, 2009). Baylis and Driver (1995) found that the visual detection of both symmetry (Experiments 1 and 2) and repetition (Experiment 4) was much harder when contour polarity mismatched. We expected to find similar results here, which would indicate the importance of contour polarity for visual regularity detection.

### Method

#### Participants

The same 24 participants who took part in Experiment 1 participated in Experiment 2 after a delay of 4 to 10 days (average 7 days). They all had normal or corrected-to-normal vision.

#### Materials and design

**Fig. 4:**
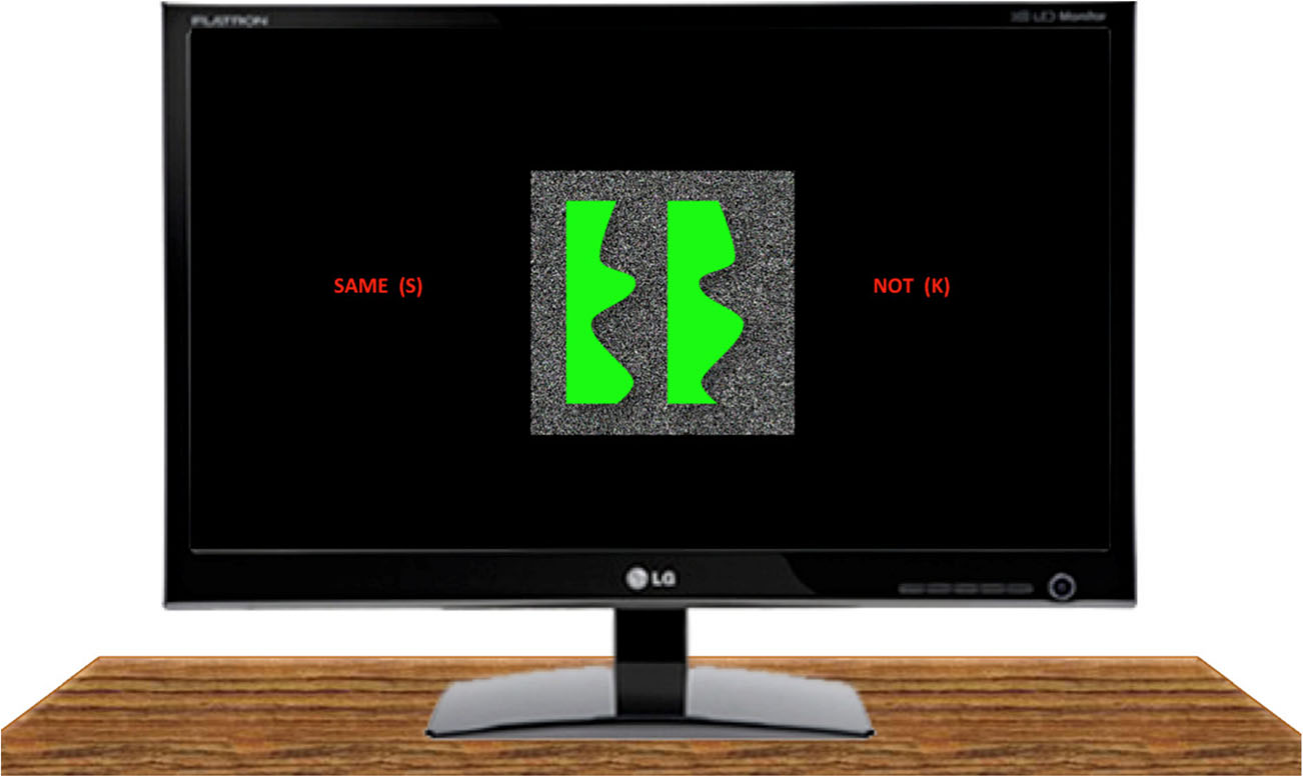
An example of a green, symmetrical, between-2objects:right-sides stimulus surrounded by a background of flickering black and white noise set within a black frame, illustrating the set-up for visual regularity detection in Experiment 2. The text flanking the stimulus reminded participants to respond with the “S” key on regular trials and the “K” key on irregular trials

The vector files used to produce the stimuli used in Experiment 1 were used to create images that were presented on a computer monitor. The monitor had a resolution of 1920 × 1080 pixels and was placed in front of, and approximately 50 cm away from, the participants’ eyes. The top of the monitor was at approximately the same height as the top of the participant’s head. Given the superior speed and accuracy of visual to haptic regularity detection, four times more trials were run in Experiment 2. In addition to the 240 stimuli used in Experiment 1, we created 240 more stimuli in the same way as in Experiment 1. These new stimuli were based on the 20 unique lines from Cecchetto and Lawson (2017) that were not used in Experiment 1. Every participant saw all 480 stimuli. The screen was black except for a centrally presented 12 cm × 12 cm background area of flickering noise. The noise consisted of squares of 2 × 2 pixels. About half of the squares were black and half were white with colour allocated at random on every frame. Objects were shown as bright green, solid surfaces (RGB: 0, 255, 0) against this background (see Fig. 4). The stimuli displayed on the monitor were matched in size to the physical stimuli used in Experiment 1, so all the stimuli were 10-cm high, and the two outer edges of the stimuli were, on average, 5 cm, 10 cm, and 7.5 cm apart for the within-1object:outer-sides, between-2objects:facing-sides, and between-2objects:right-sides stimuli, respectively. Written prompts specifying how to respond were presented on the monitor whenever the stimuli were visible (see Fig. 4). The design was identical to Experiment 1, except that each block included 240 trials for a given regularity rather than only the 60 trials used in Experiment 1. Trials were presented in a different, random order for each participant. Participants participated in the same block order (symmetry then repetition or vice versa) as they had done in Experiment 1.

#### Procedure

The procedure was identical to Experiment 1, except for the following points. Participants were instructed to centre their body midline to the centre of the monitor. The experimenter then explained the task and showed the same physical practice objects as in Experiment 1. The experiment was run using PsychoPy software (Peirce, 2007). Each block of experimental trials was preceded by 10 practice trials that were taken from that block. These practice trials were the same for all participants and they included five regular and five irregular trials, and a mixture of the three stimulus conditions. Participants were told to respond as quickly and accurately as possible using the keyboard by pressing “S” for regular stimuli and “K” for irregular stimuli. RT were recorded from the stimulus onset until the participant made a key press response. At the start of each trial, a central fixation cross appeared on the monitor for 0.5 s. This was replaced by the stimulus that remained on the monitor until the participant responded. Every 80 trials, the experiment was paused and a visual prompt appeared on the screen inviting participants to take a break. Participants resumed the experiment by pressing “G” on the keyboard. The experiment took about 30 minutes to complete.

### Results

No participants were replaced. As in Experiment 1, ANOVAs were conducted on the mean correct reaction times (RT) and percentage of errors for regular trials only, and on sensitivity (*d'*) for all trials. Correct RT faster than 0.45 s or slower than 4.5 s were discarded as outliers (less than 1.2% of trials). In the ANOVAs there were two within-participants factors: regularity type (symmetry or repetition) and condition (within-1object:outer-sides, between-2objects:facing-sides or between-2objects:right-sides). All pairwise differences noted below were significant (*p* < .05) in post-hoc Newman–Keuls analyses. Appendix 2 gives the full ANOVAs for RT, errors and sensitivity (*d'*). Here, we focus on the theoretically important effects so we only report the results for the interaction of Regularity Type × Condition and the results for the two critical comparisons.

**Fig. 5:**
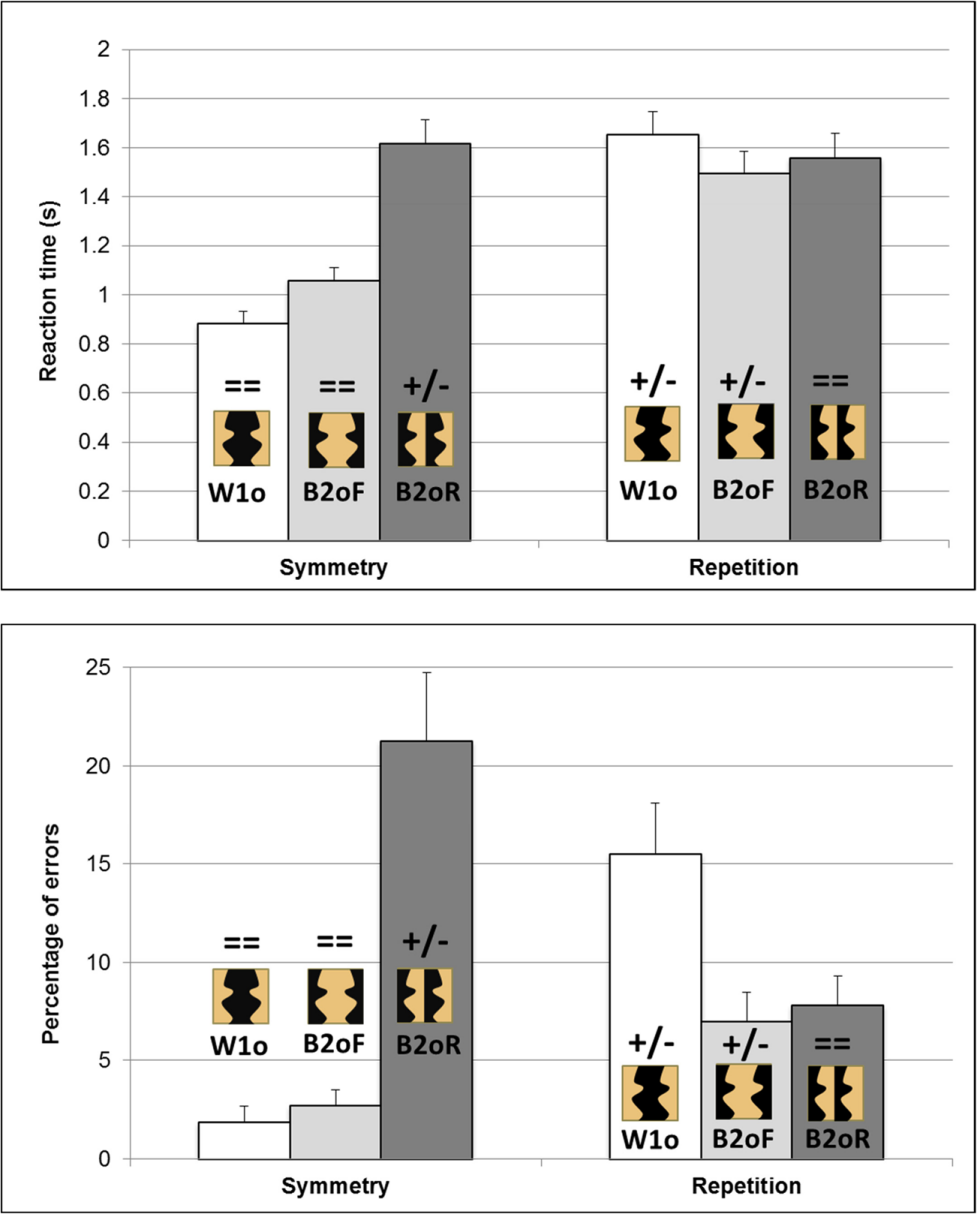
Results in Experiment 2, for regular trials, for the visual detection of symmetry and repetition, for within-1object:outer-sides (W1o; white bars), between-2objects:facing-sides (B2oF; light-grey bars), and between-2objects:right-sides (B2OR; dark-grey bars) stimuli for RT (top), and errors (bottom). Example stimuli from each condition are shown on or above each bar. For consistency with Fig. 3, these stimuli show a black object against a light brown background, but note that in Experiment 2 the objects were actually green and the background was black and white noise (see Fig. 4). Above each example stimulus, symbols indicate whether contour polarity across the pairs of critical contours matched (= =) or mismatched (+/−). Error bars represent one standard error of the mean

The interaction of Regularity Type × Condition was significant for RT, *F*(2, 46) = 61.02, *p* < .001, partial η^2^ = .73; errors, *F*(2, 46) = 31.49, *p* < .001, partial η^2^ = .58; and sensitivity *F*(2, 46) = 59.25, *p* < .001, partial η^2^ = .72 (see Fig. 5).

First, we considered whether objectness influenced visual regularity detection when contour polarity was held constant, by comparing performance for within-1object:outer-sides to between-2objects:facing-sides stimuli. For matching contour polarity stimuli, symmetry was detected faster (though no more accurately or more sensitively) for within-1object (0.88 s, 2%, *d'* of 3.79) versus between-2objects (1.06 s, 3%, 3.53) stimuli. In contrast, for mismatching contour polarity stimuli, repetition was detected slower and less accurately (though not significantly less sensitively) for within-1object (1.65 s, 16%, *d'* of 2.48) versus between-2objects (1.50 s, 7%, 2.73) stimuli. Thus, there was an interaction between objectness and regularity with opposite effects of objectness for detecting symmetry (with a within-1object:outer-sides advantage) and repetition (with a between-2objects:facing-sides advantage).

Second, we investigated whether contour polarity influenced visual regularity detection when objectness was held constant (since the critical contours always belonged to two different objects; see Fig. 1), by comparing performance for between-2objects:facing-sides to between-2objects:right-sides stimuli. Symmetry was detected faster, more accurately and more sensitively, for matching contour polarity, facing-sides stimuli (1.06 s, 3%, 3.53) than for mismatching contour polarity, right-sides stimuli (1.62 s, 21%, 2.18). Repetition was detected more sensitively (but not significantly faster or more accurately) for matching contour polarity, right-sides stimuli (1.56 s, 8%, 3.13) than for mismatching contour polarity, facing-sides stimuli (1.50 s, 7%, 2.73). Thus symmetry detection was substantially easier when contour polarity matched rather than mismatched, whereas repetition detection only showed an advantage for matching over mismatching contour polarity for sensitivity and, even there, the effect was only modest.

### Discussion

In Experiment 2 we investigated the role of objectness and contour polarity in the perception of symmetry and repetition. The same types of closed-contour, planar shapes were presented to the same participants, in the same task as in Experiment 1, but vision rather than haptics was tested.

First, visual regularity detection was influenced by objectness when contour polarity was held constant. Crucially, in contrast to haptics, objectness had the opposite effect on symmetry versus repetition detection. There was a within-1object:outer-sides advantage for symmetry detection but a between-2objects:facing-sides advantage for repetition detection (see Fig. 5). These results are consistent with previous findings from visual regularity detection but contrast to previous results for haptics (in Experiment 1 here; see also Cecchetto & Lawson, 2017; Koning & Wagemans, 2009; Lawson et al., 2016).

Second, we investigated the role of contour polarity when objectness was held constant (by considering only stimuli in which the critical contours always belonged to two different objects; see Fig. 1). As for haptics, visual regularity detection was harder when contour polarities mismatched (see Fig. 5). However, unlike haptics, this cost differed substantially depending on the type of regularity being tested. For vision, mismatching contour polarities made symmetry detection much harder but it had only a modest cost (and only for sensitivity) for repetition detection.

These two findings replicated the pattern of results obtained by Baylis and Driver (1995), who tested visual symmetry detection and visual repetition detection in separate experiments. Keeping contour polarity constant, they found a within-1object:outer-sides advantage for symmetry detection and a between-2objects:facing-sides advantage for repetition detection, with modest effects in both cases (~30–40 ms for RT, ~2% on errors, for regular trials). Keeping objectness constant, they found symmetry was much easier to detect for matching compared with mismatching contour polarities (>200 ms for RT, ~10% on errors, for regular trials) whilst repetition was somewhat easier to detect for matching compared with mismatching contour polarities (~40 ms for RT, ~2% on errors, for regular trials). Thus, consistent with our results in this experiment for visual regularity detection, Baylis and Driver (1995) observed objectness effects in opposite directions for symmetry detection and for repetition detection as well as a greater cost due to mismatching contour polarity for symmetry detection than for repetition detection. In the General Discussion, we return to consider the reasons for these differences between the visual detection of symmetry and of repetition.

## General discussion

We investigated the role of objectness and contour polarity in the detection of regularities for closed-contour, planar shapes by haptics (Experiment 1) and by vision (Experiment 2). We tested the same participants with similar sets of stimuli in both experiments. Effects of objectness (comparing pairs of critical contours belonging to opposite sides of a single object versus facing sides of two different objects) and of contour polarity (comparing stimuli with matched versus mismatched colour, luminance, concavities and convexities along pairs of critical contours) differed across the two modalities. We obtained quite similar results for symmetry detection across haptics and vision. However, we found a clear difference between the modalities for repetition detection. Cues about the distribution of objects in the external world (specifically, symmetry indicating the presence of a single object and repetition indicating the presence of multiple, similarly shaped objects) should be similar for vision and touch (Cecchetto & Lawson, 2017; Lawson et al., 2016). If regularity detection simply reflected differences in the distribution of symmetry and repetition in our physical environment then it should produce no modality-specific differences. Our results instead suggest that differences in visual versus haptic exploration, encoding and processing have powerful effects on regularity detection.

For *symmetry* detection, both haptics and vision had greater sensitivity to contour polarity (comparing between-2objects:facing-sides to between-2objects:right-sides stimuli, where objectness was held constant) than to objectness (comparing within-1object:outer-sides to between-2objects:facing-sides stimuli, where contour polarity was held constant). Symmetry was much harder to detect when contour polarity mismatched for both haptics and vision. In contrast, objectness had no effect for haptics, and only a modest effect for vision (with a within-1object:outer-sides advantage for detection speed only).

For *repetition* detection, haptics had greater sensitivity to contour polarity than to objectness, with performance similar to that of both haptic and visual symmetry detection. Haptic repetition detection was much harder when contour polarity mismatched, whilst there was only a modest effect of objectness (with a within-1object:outer-sides advantage for detection speed only). In contrast, visual repetition detection showed strikingly different effects. First, mismatching contour polarity produced no cost on speed or accuracy and only a modest cost on sensitivity. Second, objectness had a powerful effect that was in the opposite direction to the three other conditions (with a between-2objects:facing-sides advantage).

In this study, participants were told which regions to interpret as objects, and which as background. Participants may, though, have undertaken a figure-ground reversal. For example, the between-2objects:facing-sides stimuli may have been perceived with the central, background region as the object flanked by two background regions. If so, they would have been interpreted in the same way as the within-1object:outer-sides stimuli. Two pieces of evidence argue against this possibility. First, performance differed significantly across several objectness conditions, indicating that people interpreted them differently (for haptic repetition detection in Experiment 1, see Figure 3, and for visual symmetry and repetition detection in Experiment 2; see Fig. 5). Second, instructions such as we provided have been found to suffice to determine what regions are interpreted as objects (Baylis & Driver, 1995, Experiment 3). In a symmetry detection task, Baylis and Driver told participants to assign red surfaces as figure and green surfaces as background, or vice versa. They found that performance differed for physically identical stimuli depending on the instructions given. The present study used a similar task and stimuli to Baylis and Driver (1995). Furthermore, unlike their stimuli, there were salient physical differences between the figure and the background regions that reduced the ambiguity of figure-ground assignment. For haptics, in Experiment 1, the smooth, plastic, 3-D planar objects were raised 5 mm above the background surface of cardboard. For vision, in Experiment 2, the smooth, solid, bright surface of the object was surrounded by a background of dim, flickering noise.

Across a series of related studies (here, and in Cecchetto & Lawson, 2017; Lawson et al., 2016), we have tested several cues which seem to be important for defining what is an object for vision and haptics. This is an ambitious topic to tackle, given that it has proven difficult to provide a formal definition of objectness for vision (Feldman, 2003), whilst for haptics we are not aware that this topic has even been discussed before. Other studies that have investigated the effects of objectness on regularity detection (e.g., Baylis & Driver, 1995, 2001; Bertamini, 2010; Bertamini et al., 1997; Corballis & Roldan, 1974; Koning & Wagemans, 2009; Treder & van der Helm, 2007) have not usually discussed how they defined objects. These authors used a diverse range of visual cues (e.g., contour closure, colour, luminance, type of regularity, stratification of surfaces in depth, line and dot separation, verbal instructions) to try to manipulate what is perceived as an object. However, in many cases, these manipulations, in turn, introduced confounds (such as differences in luminance), which meant that within-1object and between-2objects stimuli differed in important respects other than objectness. For example, as noted by van der Helm and Treder (2009), contour polarity often matched in some conditions and mismatched in others. This difficulty in producing stimuli to use to test the effects of objectness means that no single approach is likely to allow watertight conclusions to be drawn about its role. Given this, we argue that the best approach is to attempt to find converging evidence by systematically varying multiple factors to investigate objectness, including using different modalities, different tasks, different modes of exploration, and different stimuli. This was our aim in the present experiments.

**Fig. 6:**
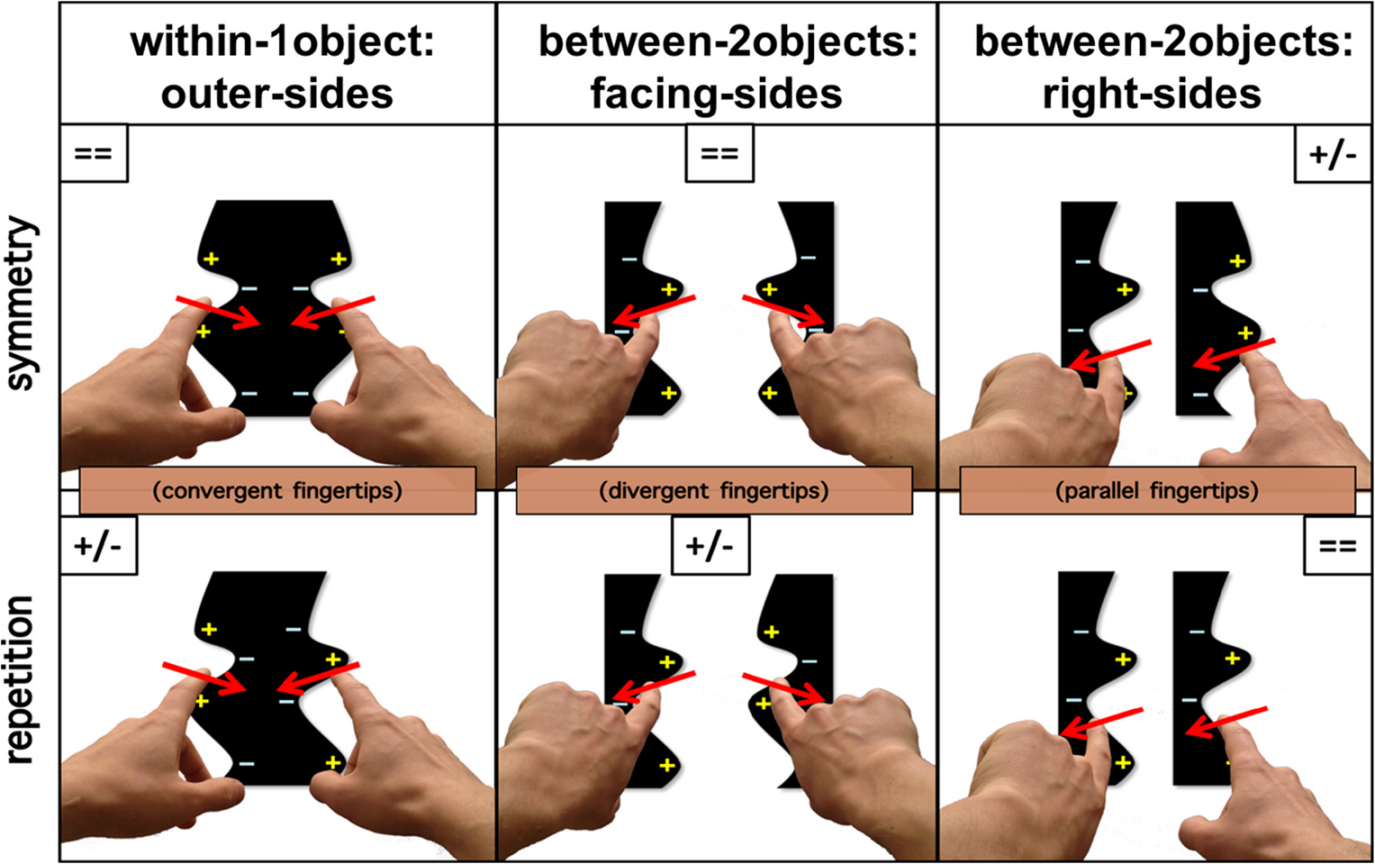
How the six different conditions for regular stimuli tested in Experiment 1 might be explored haptically. Objects are shown as black, closed-contour shapes against a white background space. For within-1object:outer-sides stimuli (first column) and between-2objects:facing-sides stimuli (second column), symmetry detection might be easier than repetition detection due to the manner of exploration. The preferred exploration of symmetrical stimuli would be symmetrical for these stimuli (with convergent movements, pressing the fingers together, for within-1object:outer-sides stimuli and divergent movements, pushing the fingers apart, for between-2objects:facing-sides stimuli, see the red arrows). In contrast, these symmetrical exploration movements would be incongruent with the type of regularity to be detected for repeated stimuli. The reverse pattern would be expected for between-2objects:right-sides stimuli (third column). Here, repetition detection might be easier than symmetry detection because people would naturally explore these stimuli using repeated, parallel movements

One factor that may be particularly important for detecting haptic regularities, and for haptically defining objects, is the manner of stimulus exploration. For vision there has been some work on the interactions between effects of exploration and the manner of information extraction and perception. For example, eye movements can influence shape perception for 3-D objects (e.g., Leek et al., 2012) and the presence of symmetry can influence eye movements (e.g., Kootstra, de Boer, & Schomaker, 2011; Locher & Nodine 1973; Meso, Montagnini, Bell, & Masson, 2016). Other work has tested the effect of making visual exploration more like that of haptics, for example by using aperture viewing (Lawson & Cecchetto, 2018; Martinovic, Lawson, & Craddock, 2012). For haptics, we have found that varying whether one hand or two hands are used to feel stimuli influences the detection of regularities (Cecchetto & Lawson, 2017; Lawson et al., 2016). In Experiment 1, here, participants always used the index fingers of their right and left hands to explore the right and left critical contours, respectively, so the same gross hand movements were always used. However, from observing participants performing the task, we believe that exploration differed across the three stimulus conditions, with the 3-D structure of the stimuli encouraging the fingertips to be directed at different orientations (see the red arrows in Fig. 6). These directions were symmetrically convergent, symmetrically divergent or repeatedly parallel during the exploration of within-1object:outer-sides, between-2objects:facing-sides and between-2objects:right-sides stimuli, respectively.

Stimulus-driven differences in the mode of haptic exploration may have enhanced effects of contour polarity on regularity detection for haptics relative to vision. We propose that haptic regularity detection is easy when the regularity being detected (symmetry vs. repetition) is congruent with how the stimulus is being explored (matched converging and diverging movements for symmetry; parallel movements for repetition). In future research we intend testing this hypothesis directly, by requiring participants to feel contours from a particular direction, in order to manipulate whether exploration favours the detection of symmetry versus repetition. If haptic regularity detection is directly influenced by the movements used during exploration this would provide further support for our general claim that regularity detection depends on how we acquire and process information.

A final point is that participants explored our haptic stimuli using contour following. This exploratory procedure is usually used to extract local shape information and is the normal movement used to feel the shape of planar stimuli (Lawson & Bracken, 2011). In contrast, global shape information is usually extracted by enclosing an object using the whole hand (Lederman & Klatzky, 1987). We have no reason to expect that our results would differ for nonplanar objects being explored using enclosure as well as contour following or for tasks with shorter exploration times. However, these situations remain to be tested.

In conclusion, for both vision and haptics, we found that symmetry detection was harmed more by having to compare contours with mismatching polarity than contours belonging to two different objects. For haptics, repetition detection was again harmed more by mismatching contour polarity than by making comparisons across two different objects. However, the results were very different for visual regularity detection. Here, first, there was a substantial *benefit* to comparing contours across two different objects (relative to when both contours belonged to the same object). Second, the within-1object cost for visual regularity detection outweighed the cost of mismatching contour polarity. These two differences between visual and haptic repetition detection support our wider claim that vision and haptics differ in how they weight the cues that they use to determine the presence and location of objects (see also Cecchetto & Lawson, 2017; Lawson et al., 2016). We further speculate that contour polarity may be particularly important for haptics due to how we move our hands as we feel the edges of objects (see Fig. 6). Our findings of modality-specific differences in regularity detection show that how we perceive symmetry and repetition does not solely reflect what physical information about objects is available in our environment. Instead we believe that effects on regularity detection may primarily reflect how we extract and use information in vision and haptics.

## Appendix 1

The full ANOVAs for RT, errors and sensitivity (*d'*) for haptic regularity detection in Experiment 1 are presented here. In the ANOVAs there were two within-participants factors: regularity type (symmetry or repetition) and condition (within-1object:outer-sides, between-2objects:facing-sides or between-2objects:right-sides). All pairwise differences noted below were significant (*p* < .05) in post hoc Newman-Keuls analyses.

Regularity-type was significant for RT, *F*(1, 23) = 4.97, *p* = .036, partial η^2^ = .18, and sensitivity, *F*(1, 23) = 4.81, *p* = .039, partial η^2^ = .17, but not for errors, *F*(1, 23) = 2.14, *p* = .157, partial η^2^ = .09. Condition was significant for RT, *F*(2, 46) = 11.86, *p* < .001, partial η^2^ = .34; errors, *F*(2, 46) = 13.10, *p* < .001, partial η^2^ = .36; and sensitivity, *F*(2, 46) = 37.93, *p* < .001, partial η^2^ = .62. Most importantly, the interaction of Regularity Type × Condition was significant for RT, *F*(2, 46) = 34.86, *p* < .001, partial η^2^ = .60; errors, *F*(2, 46) = 90.47, *p* < .001, partial η^2^ = .79; and sensitivity, *F*(2, 46) = 28.10, *p* < .001, partial η^2^ = .55 (see Fig. 3).

First, comparing the different conditions for each regularity type in turn, for symmetry detection, both within-1object:outer-sides (7.1 s, 3%, *d'* of 2.08) and between-2objects:facing-sides (8.1 s, 5%, 1.87) stimuli were detected faster, more accurately and more sensitively than between-2objects:right-sides stimuli (11.7 s, 67%, 0.19), with no difference between them. In contrast, for repetition detection, nearly the opposite pattern of results was obtained. Here, both within-1object:outer-sides (10.0 s, 39%, 1.05) and between-2objects:facing-sides (11.9 s, 46%, 0.95) stimuli were detected slower and less accurately than the between-2objects:right-sides stimuli (8.5 s, 3%, 1.20), with the same trend for sensitivity, though the differences there were not significant in post hoc Newman–Keuls analyses. Also, within-1object:outer-sides repeated stimuli were detected faster (but not more accurately or more sensitively) than between-2objects:facing-sides repeated stimuli. Second, comparing the two types of regularity for each condition in turn, symmetry with matching contour polarities was detected faster, more accurately, and more sensitively than repetition with mismatching contour polarities for both within-1object:outer-sides stimuli and between-2objects:facing-sides stimuli, whilst the reverse held for between-2objects:right-sides stimuli: Here, symmetry with mismatching contour polarities was harder to detect than repetition with matching contour polarities.

## Appendix 2

The full ANOVAs for RT, errors and sensitivity (*d'*) for visual regularity detection in Experiment 2 are presented here. In the ANOVAs there were two within-participants factors: regularity type (symmetry or repetition) and condition (within-1object:outer-sides, between-2objects:facing-sides, or between-2objects:right-sides). All pairwise differences noted below were significant (*p* < .05) in post hoc Newman–Keuls analyses.

Regularity type was significant for RT, *F*(1, 23) = 26.43, *p* < .001, partial η^2^ = .54, and sensitivity *F*(1, 23) = 17.54, *p* < .001, partial η^2^ = .43, but not for errors, *F*(1, 23) = 1.44, *p* = .24, partial η^2^ = .06. Condition was significant for RT, *F*(2, 46) = 48.49, *p* < .001, partial η^2^ = .68; errors, *F*(2, 46) = 18.05, *p* < .001, partial η^2^ = .44; and sensitivity, *F*(2, 46) = 19.54, *p* < .001, partial η^2^ = .46. Most importantly, the interaction of Regularity Type × Condition was significant for RT, *F*(2, 46) = 61.02, *p* < .001, partial η^2^ = .73; errors, *F*(2, 46) = 31.49, *p* < .001, partial η^2^ = .58; and sensitivity, *F*(2, 46) = 59.25, *p* < .001, partial η^2^ = .72 (see Fig. 5).

First, comparing the different conditions for each regularity type in turn, for symmetry detection, both within-1object:outer-sides (0.88 s, 2%, *d'* of 3.79) and between-2objects:facing-sides (1.06 s, 3%, 3.53) stimuli were detected faster, more accurately, and more sensitively than between-2objects:right-sides stimuli (1.62 s, 21%, 2.18). In addition, within-1object:outer-sides stimuli were detected faster than between-2objects:facing-sides stimuli. Thus, for symmetry detection, there was a similar pattern of results for vision and haptics. For repetition detection, within-1object:outer-sides stimuli (1.65 s, 16%, *d'* of 2.48) were detected slower and less accurately (but not significantly less sensitively) than between-2objects:facing-sides stimuli (1.50 s, 7%, 2.73) and less sensitively (but not significantly slower or less accurately) than between-2objects:right-sides stimuli (1.56 s, 8%, 3.13). In addition, between-2objects:facing-sides stimuli were detected less sensitively (but not significantly slower or less accurately) than between-2objects:right-sides stimuli. Second, comparing the two types of regularity for each condition in turn, symmetry with matching contour polarities was detected faster, more accurately and more sensitively than repetition with mismatching contour polarities for within-1object:outer-sides stimuli. Similarly, symmetry with matching contour polarities was detected faster and more sensitively (though not significantly more accurately) than repetition with mismatching contour polarities for between-2objects:facing-sides stimuli. In contrast, symmetry with mismatching contour polarities was detected less accurately and less sensitively (though not significantly faster) than repetition with matching contour polarities for between-2objects:right-sides stimuli.
